# Berberine ameliorates septic cardiomyopathy through protecting mitochondria and upregulating Notch1 signaling in cardiomyocytes

**DOI:** 10.3389/fphar.2024.1502354

**Published:** 2024-11-06

**Authors:** Qi Shen, Yufan Yuan, Zelin Li, Ying Ling, Jian Wang, Mingjing Gao, Peng Wang, Mengli Li, Lizhong Lai, Jinlan Jin

**Affiliations:** ^1^ Department of Critical Care Medicine, Shenzhen Hospital (Futian) of Guangzhou University of Chinese Medicine, Shenzhen, China; ^2^ Department of Basic Research of Integrated Traditional Chinese and Western Medicine, Guangzhou University of Chinese Medicine, Guangzhou, China; ^3^ Department of Automation, Tsinghua University, Beijing, China; ^4^ Department of Pathology, Shenzhen Hospital (Futian) of Guangzhou University of Chinese Medicine, Shenzhen, China

**Keywords:** septic cardiomyopathy, mitochondria, Notch1 signaling pathway, berberine, cardiomyocytes

## Abstract

**Introduction:**

Septic cardiomyopathy (SCM) arises as a consequence of sepsis-associated cardiovascular dysfunction, for which there is currently no specific targeted therapy available. Previous studies have demonstrated the beneficial therapeutic effect of berberine (BBR) on SCM; however, the underlying mechanisms of action remain unclear. The objective of this is to elucidate how BBR alleviates SCM.

**Methods:**

Septic cardiomyopathy rat model was established by performing cecal ligation and puncture (CLP), while a cardiomyocyte injury model was provoked in H9C2 cells using lipopolysaccharide (LPS). Cardiac function was assessed through echocardiography, and myocardial histopathology was examined with hematoxylin-eosin (HE) staining. Cardiomyocyte viability was determined through Cell Counting Kit-8 (CCK8) assay, and measurement of ATP levels was done with an ATP assay kit. Mitochondrial ultrastructure was observed using transmission electron microscopy. Real-time polymerase chain reaction (RT-PCR) and Western blotting were employed to analyze the expression of Notch1 signaling pathway components and downstream molecules in myocardial tissues and cells.

**Result:**

*In vivo*, BBR markedly improved symptoms and cardiac function in SCM rats, leading to enhanced ATP content, and ameliorated mitochondrial structure. Additionally, BBR increased Notch1 protein expression in myocardial tissue of the rats. *In vitro*, BBR elevated the survival rates of H9C2 cell, improved mitochondrial morphology, and raised ATP levels. The mRNA expression of Notch1, Hes1, and Hes2, and Notch1 protein expression was upregulated by BBR. While these effects were reversed upon inhibiting the Notch1 signaling pathway.

**Conclusion:**

BBR improves septic cardiomyopathy by modulating Notch1 signaling to protect myocardial mitochondria.

## 1 Introduction

Septic cardiomyopathy (SCM), a severe complication arising from sepsis, poses significant threats to cardiac health as part of the multi-organ dysfunction triggered by an aberrant immune response to infections (Ravikumar et al., 2021). This condition is embedded within the intricate pathology of sepsis, characterized by a tangled web of inflammation, disturbed mitochondrial function, oxidative stress, and immune system imbalance (Wang et al., 2022). Sepsis-induced myocardial dysfunction, known as sepsis cardiomyopathy (SCM), is a common and serious complication with high mortality rates ranging from 70% to 90% in patients with SCM compared to 20% in those without SCM (Steven and Mervyn, 2021). Currently, clinical treatment strategies primarily focus on supportive care, including fluid resuscitation, vasopressor agents, and supportive mechanical ventilation. While these measures can improve patient survival rates to some extent, the mortality rate remains alarmingly high, presenting a significant challenge in the field of critical care medicine (Huan et al., 2020). Existing treatment strategies have limited efficacy in addressing the underlying causes, particularly mitochondrial dysfunction and inflammatory responses (Levy et al., 2018). Now, the guidelines for sepsis treatment have been updated, mentioning the use of vasopressors and their potential side effects. Approximately 25% of sepsis patients receiving high-dose norepinephrine developed arrhythmias, 15% experienced myocardial ischemia, and the incidence of acute kidney injury was 40% (Shapiro et al., 2023; Qin et al., 2024). There is a critical need to develop new and effective drugs to prevent and mitigate myocardial damage in SCM.

Within the context of SCM, the preservation of mitochondrial structure and function in cardiomyocytes is fundamental to maintaining cardiac fitness and ensuring cell viability. Extensive research reveals decreased activity of mitochondrial genes and impaired mitochondrial morphology in SCM patient hearts (Hollenberg and Singer, 2021; Zhou et al., 2023). This includes focal disruptions to the mitochondrial membrane, swelling, and distortions of the cristae (Zhou et al., 2023; Li et al., 2024). Such anomalies, compounded by an inadequacy in mitochondrial biosynthesis and an overzealous process of mitochondrial fission, conspire to undermine cardiac function, precipitating cardiomyocyte demise and a precipitous decline in cardiac output (Feng et al., 2019; L'Heureux et al., 2020; Liu et al., 2017). This highlights mitochondrial dysfunction as a pivotal event in the pathophysiology of SCM, where it not only curtails blood flow to the myocardium but also acts as a pivotal trigger for multi-organ failure, thereby exacerbating the grim prognosis faced by sepsis patients (Martin et al., 2019; Stanzani et al., 2019). Thus, preserving mitochondrial health and balancing their structural functionality are central strategies for both treating and preventing SCM.

The Notch signaling pathway, a fundamental communicator between cells, is widely influential in various biological settings due to its strong conservation (Stanzani et al., 2019; Chaves et al., 2018). Through a meticulously orchestrated sequence of events initiated by ligand-receptor interaction and subsequent proteolytic processing, the Notch intracellular domain (NICD) is released to engage the transcription machinery, orchestrating a cascade of downstream effects through target genes like Hes and Hey (Kasahara et al., 2013). In the cardiac context, Notch signaling is vital for heart cell survival, growth from precursor cells, and blood vessel formation (MacGrogan et al., 2018), highlighting its key role in heart health and healing. Emerging evidence positions Notch1 signaling protects the heart from damage, effective in combating heart disease, heart attacks, abnormal growth, and injury from restored blood flow (Meester et al., 2019; Feng et al., 2023), as activation of Notch signaling effectively mitigates ischemic injury, protects cardiomyocytes from apoptosis, and regulates cardiac self-repair after myocardial infarction. Targeting the Notch pathway presents novel therapeutic avenues for SCM, harnessing its potential to revert detrimental mitochondrial alterations associated with this condition (Parmigiani et al., 2022).

Berberine (BBR), a potent alkaloid extracted from plants (Shihai et al., 2022), is considered one of the most biologically active compounds found in Berberis species (Yalan et al., 2022). According to traditional Chinese medicine, BBR is believed to possess properties that clear the fire of heart, and detoxify the body (western medicine thinks it can eliminate inflammation), making it a commonly used treatment for infectious diseases (Danyang et al., 2020). Modern pharmacological studies have further confirmed its diverse effects, including anti-tumor, hypoglycemic and cardiovascular and cerebrovascular protective, anti-inflammatory, anti-Alzheimer’s disease, antiarrhythmic and antidepressant properties (Erjie et al., 2023; Shuangyuan et al., 2022). In the treatment of septic cardiomyopathy (SCM), berberine (BBR) demonstrates potential advantages in anti-inflammatory, antioxidant, and mitochondrial function regulation, addressing the limitations of current SCM treatment strategies in effectively managing the underlying causes (Xu et al., 2019; Xu et al., 2020).

Recent studies have found that BBR alleviates sepsis-induced cognitive impairment and improved survival rates in septic mice (Jian et al., 2021). Our previous investigation also revealed that BBR intervention enhanced cardiac systolic and diastolic function, attenuated cardiomyocyte injury and inflammatory cell infiltration in sepsis rats (Huiqi et al., 2021). Berberine (BBR) has proven efficacious in diverse contexts, demonstrating mitigation of chronic intermittent hypoxia-induced cardiac damage through mitochondrial biogenesis stimulation and protection against ischemia-reperfusion injury by maintaining mitochondrial integrity (Kasahara et al., 2013; Chen H. et al., 2021). Its regulation of mitochondrial activity, which alleviates myocardial ischemia-reperfusion harm, introduces a new therapeutic angle (MacGrogan et al., 2018). These findings underscore BBR’s potential in both preventing myocardial injury and restoring mitochondrial function, highlighting the need for focused research on its mechanisms and efficacy in septic cardiomyopathy (SCM). But there are also limitations in the treatment of sepsis, including restrictions on indications, issues with drug distribution and metabolism, and the need for further research on its long-term efficacy and safety (Lv et al., 2023; Ai et al., 2021). The pharmacokinetic properties of berberine (BBR), including absorption, distribution, metabolism, and excretion, determine its safety and efficacy. However, BBR has low oral bioavailability, approximately 5% (Chen HB. et al., 2021), limiting its clinical effectiveness. Recent research shows that nanotechnology, such as nanocarriers, liposomes, and microfluidic-assisted methods, can significantly enhance BBR’s bioavailability. These systems optimize drug absorption and distribution, improving efficacy and safety (Dewanjee et al., 2020). Nanocarriers protect BBR from degradation by gastric acid and enzymes, enhancing intestinal absorption. Liposomes facilitate BBR delivery into target cells through passive diffusion or endocytosis. Microfluidic technology controls drug release rates, further boosting bioavailability and therapeutic outcomes (Guo et al., 2019). These innovations offer new ways to enhance BBR’s clinical efficacy, potentially overcoming its low bioavailability and maximizing its role in treating septic cardiomyopathy (SCM).

Despite our previous *in vivo* study showing that BBR can inhibit the sepsis-induced TLR4/NF-κB signaling pathway (Chen H. et al., 2021), the mechanism of BBR’s treatment of SCM is very complex and requires further research, particularly regarding the role of BBR in SCM. Existing research are short of adequately explored the specific mechanisms by which berberine acts in septic cardiomyopathy (SCM), and insufficient understanding of how the Notch signaling pathway regulates mitochondria. Therefore, we investigated the effect of BBR on Notch signaling and mitochondria by replicating the SCM model in rats and H9C2 cells, and explored the therapeutic effect of BBR on SCM and the specific targets generated by its effects, in order to provide experimental basis for the clinical application of BBR in the prevention and treatment of SCM.

## 2 Materials and methods

### 2.1 Cell culture and processing

The H9C2 line was purchased from the ventricular cardiomyocyte line of Wuhan Prosel Life Science and Technology Co., Ltd. Cells were cultured in basal high-glucose medium (DMEM, Wuhan Proxel Technology Co., Ltd.) containing 10% fetal bovine serum (Wuhan Proxel Technology Co., Ltd.) and 1% penicillin/streptomycin (Wuhan Proxel Technology Co., Ltd.). Place in a normal cell culture incubator for culture. The normal group (Con group), model group (LPS group), administration group (LPS + BBR group) and inhibitor group (DAPT + LPS + BBR group) were set up. Lipopolysaccharide (LPS, L2630, Sigma) (10 μg/mL) was used to induce H9C2 cell damage (Sheehan et al., 2022; Page et al., 2022), and BBR (50 μmol/L) was given for intervention (Liang et al., 2019). In the inhibitor group, Notch1 signal blocker (DAPT, SF4139, Biyuntian) (50 μmol/L) was used (Zhang et al., 2015), and cardiomyocytes were observed after 24 h for follow-up experiments.

### 2.2 Cell viability analysis

Cell viability was measured using the Cell Counting Kit-8 assay (CCK-8, Biotech Institute, Shanghai, China). Seed the cell suspension (100 μL/well) in a 96-well plate. Pre-incubate in a humidified incubator for a certain amount of time (37°C, 5% CO2). Add 10 μL of CCK-8 solution to each well of the plate according to the instructions provided by the manufacturer and place the plate in the incubator for 1–4 h. Measure the absorbance of each well using an ELX808 microplate reader (Apbay Biotechnology (Suzhou) Co., Ltd.) at 450 nm. Viability levels of H9C2 cells are normalized to a ratio of control groups.

### 2.3 Animal experiments

All operations in this study are in accordance with the approval of the Animal Experiment Ethics Committee of Guangzhou University of Chinese Medicine (20,220,228,058). Sixty male Sprague-Dawley rats (200 ± 20 g) used in this study were obtained from Guangzhou Ruige Biotechnology Co. (animal qualification number: SCXK 2021–0059). The rats were housed in a 12 h light/12 h no light environment under Specific Pathogen Free (SPF) conditions, the laboratory environment temperature was maintained between 24 and 26°C, the relative humidity was kept in the range of 50%–70%, ventilation was maintained, 4 rats/cage were raised, in the process of adaptive feeding for 1 week, the rats could eat and drink freely, and the corn cob bedding was changed regularly. Fasting and non-water fasting prior to the start of the experiment, and all rats are randomly numbered, weighed, and recorded. The rats were divided into four groups (n = 20) using the numeric random table method: sham operation (Sham) group; Sepsis model (CLP) group; BBR hydrochloride (Shanghai Macklin, B802465, purity ≥98%) intervention (CLP + BBR) group; Sham operation plus BBR hydrochloride intervention group (Sham + BBR) group. To evaluate the successful establishment of the SCM model in rats, cardiac functional parameters were assessed at 24 h and 48 h post-modeling. Heart tissue morphology was examined through HE staining (Chen H. et al., 2021).

### 2.4 Treatment

The rats were divided into 4 groups (n = 20) using the numeric random table method: sham operation (Sham) group (open and closed abdomen, no ligation and puncture of the cecum; ddH2O, 1 mL/100 g, gavage, q12 h); CLP group (cecal ligation perforation; ddH2O, 1 mL/100 g, gavage, q12 h); CLP + BBR group (cecal ligation perforation; BBR, 50 mg/kg, gavage, q12 h) (Li et al., 2023); Sham + BBR group (open and closed abdomen, no ligation and puncture of the cecum; BBR, 50 mg/kg, gavage, q12 h). A rat model of sepsis was established using the CLP method (Atsuko et al., 2013; Donal et al., 2018). Rats were intraperitoneally injected with 4% sodium pentobarbital (obtained from Beijing Ouhua Technology Co., Ltd., OH004809, purity 98%, dosage 0.2 mL/kg) to induce anesthesia. Disinfect the abdomen with alcohol after shaving. After the laparotomy, 50% of the cecum is ligated with non-absorbable sutures, two holes are pierced with a 20 G syringe needle, part of the intestinal contents are squeezed out, and the abdominal cavity is put back into the abdominal cavity to close the wound. The sham group underwent laparotomy and did not have CLP. BBR hydrochloride is treated with intragastric administration (50 mg/kg, q12, dose selection was based on literature support and the preliminary experimental exploration of our research group). Rats are rapidly resuscitated by subcutaneous injection of normal saline (50 mg/kg). Flurbiprofen cilofen ester (Shanghai Macklin, B802465, purity ≥98%) injection 5 mg/kg intravenous injection was used for relief.

### 2.5 Echocardiography

After intraperitoneal injection of 4% sodium pentobarbital (0.2 mL/kg), the rats were anesthetized and their abdominal hair was removed. Echocardiographic images were recorded using color Doppler echocardiography of the small animal heart (Visual Ultrasound Vevo 2,100 Imaging System). Cardiac function parameters were assessed in a parasternal long-axis view in 2D mode before m-mode images perpendicular to the ventricular septum and the posterior wall of the left ventricle were obtained. Heart rate is calculated for three consecutive heart cycles. Left ventricular stroke volume (SV), left ventricular ejection fraction (LVEF), cardiac output (CO), and left ventricular shortening fraction (LVFS) were calculated using software for the Vevo770TM imaging system. End-systolic left ventricular anterior wall thickness (LVAWs), left ventricular anterior wall diameter (LVIDd), end-diastolic left ventricular anterior wall thickness (LVAWd), and left ventricular end-systolic diameter (LVIDs) were recorded. All data were tested and calculated using the methods proposed by the American Society of Echocardiography (Sherif et al., 2016). The experiment was performed by an experimenter with prior knowledge of the experimental procedure. At the end of the experiment, the rats were euthanized under deep anesthesia. Tissue and blood samples were collected for further analysis.

### 2.6 Hematoxylin and eosin (HE) staining

After the rats were sacrificed, the myocardial tissue at the apex of the heart was taken and fixed with 4% neutral formaldehyde solution. The embedded sections were then stained and images were collected at 400 × magnification under a light microscope (Obus, CX22).

### 2.7 Transmission electron microscopy

Rats are cardiac perfused with normal saline and 2% glutaraldehyde. Approximately 1 mm^3^ of cortical tissue was collected and fixed in glutaraldehyde. The tissue is embedded in epoxy resin and sectioned. Sections (0.12 μm) are stained with 1% uranyl acetate. Mitochondrial ultrastructure was observed by transmission electron microscopy (Hitachi, HT7800).

### 2.8 ATP test

An enhanced ATP assay kit from Beyond (Shanghai, China) was used to quantify the amount of ATP in rat myocardial tissue with H9C2 cells according to the manufacturer’s instructions. The luminescence signal is used to calculate the total ATP level, which is then normalized to the protein concentration.

### 2.9 Immunofluorescence

Immunostaining was conducted using primary antibody against Notch1 (1:15 dilution, Abcam, United Kingdom), followed by secondary Goat Anti-Rabbit IgG (H + L)-Cy3 conjugated (1:20 dilution, affinity, United States). Nuclei were stained with DAPI (Beyuntian, China) and imaged under a ZOETM fluorescent microscope (BIO-RAD, United States).

### 2.10 Real-time polymerase chain reaction

Total RNA is isolated from rat myocardial tissue or H9C2 cells with RNA extraction kit (Solarbio, R1200). The isolated RNA was reverse transcribed with a kit from AG Biotechnology (Hunan, China AG11728). The SYBR Green Pro Taq HS premixed qPCR kit from China AG Biotechnology Co., Ltd. (Hunan, AG11701) was used. Expression of specific genes was detected and analyzed using BIO-RAD CFX96 (BIO-RAD, United States). cDNA was extracted from RNA using primers according to the instructions of the Evo M-MLV Reverse Transcription Master Mix Kit, and all cDNA was stored at −80°C before use. The PCR tube containing the cDNA reaction was placed in BIO-RAD CFX96 for amplification, and the reaction conditions were as follows: pre-denaturation 95°C for 30 s, denaturation 95°C for 30 s Annealing 60°C for 30 s, elongation 72°C for 40 s, reading plate at 80°C. Set the above denaturation-annealing-elongation to 40 cycles. After the amplification curves were analyzed, the gene expression was calculated by 2^−ΔΔCT^ method and glyceraldehyde-3-phosphate dehydrogenase (GAPDH) as the internal control. Primer sequence records are shown in Table 1.

**TABLE 1:** Primer sequences for PCR.

Notch1	F: 5′-GCCAGCAAGAAGAAGCGGAGAG-3′ R: 5′-CCACTCGTTCTGATTGTCGTCCATC-3′
Hes1	F: 5′-CTAACGCAGTGTCGCCTTCCAG-3′ R: 5′-AGAGAGGTGGGCTAGGGAGTTTATG-3′
Hes2	F: 5′-CCTGAAGCCACTGCTGGAGAAG-3′ R: 5′-AGCGGCAATACTAGACCCTTTAGC-3′
Hey1	F: 5′-GGCTATGGACTATCGGAGTTTGGG-3′ R: 5′-AGGCGAACACGAAGCGGATC-3′
Hey2	F: 5′-CGGGAGGCAGCAGTGATGAC-3′ R: 5′-ATAGGCGACATGGCGTTGACTC-3′
GAPDH	F: 5′-ACGGCAAGTTCAACGGCACAG-3′ R: 5′-CGACATACTCAGCACCAGCATCAC-3′

### 2.11 Western blot detection

Frozen myocardial tissue or H9C2 cells are rapidly thawed, homogenized, and lysed in RIPA buffer (China) containing protease and phosphatase inhibitors (Beyotime, Shanghai, China) to denature the proteins. Protein samples (50 μg) were first added to SDS-PAGE for electrophoresis and then transferred to PVDF membranes. Block the membrane with blocking solution for 1 h, followed by Notch1 antibody (1:1,000 dilution, Abcam, AM, Cambs, United Kingdom), NICD antibody (1:1,000 dilution, affinity, United States). Incubate overnight at 4°C. The membrane was washed three times with wash solution, followed by incubation with goat anti-mouse antibody (1:5,000 dilution, Boster, Wuhan, China) for 1 h. After thorough washing with washing solution, observe the immune response bands using Tanon4600. Using ImageJ, protein expression was quantified based on band intensity.

### 2.12 Statistical analysis

Statistical analysis was performed using SPSS26.0 software. The experimental data were tested by Shapiro-wilk for normality. Descriptive statistics were presented as mean ± standard deviation (SD) for normally distributed data, and as median (P25–P75) for skewed data. For data comparison between multiple groups, one-way ANOVA was used for those with normal distribution and homogeneity of variance, LSD method was used for multiple comparison, Welch test was used for data with uneven variance, and Dunnett'3 test was onducted for multiple comparison. For non-normally distributed data, the nonparametric rank-sum test (Kruskal Wallis H) was conducted for multiple samples. A significance level of P< 0.05 was considered statistically significant. Graphical representations of the statistical results were generated using GraphPad Prism version 8 software (GraphPad, San Diego, CA, United States), with histograms used for normally distributed data and box plots for non-normally distributed datasets.

## 3 Results

### 3.1 BBR improves cardiac function and myocardial tissue damage in sepsis rats

**FIGURE 1:**
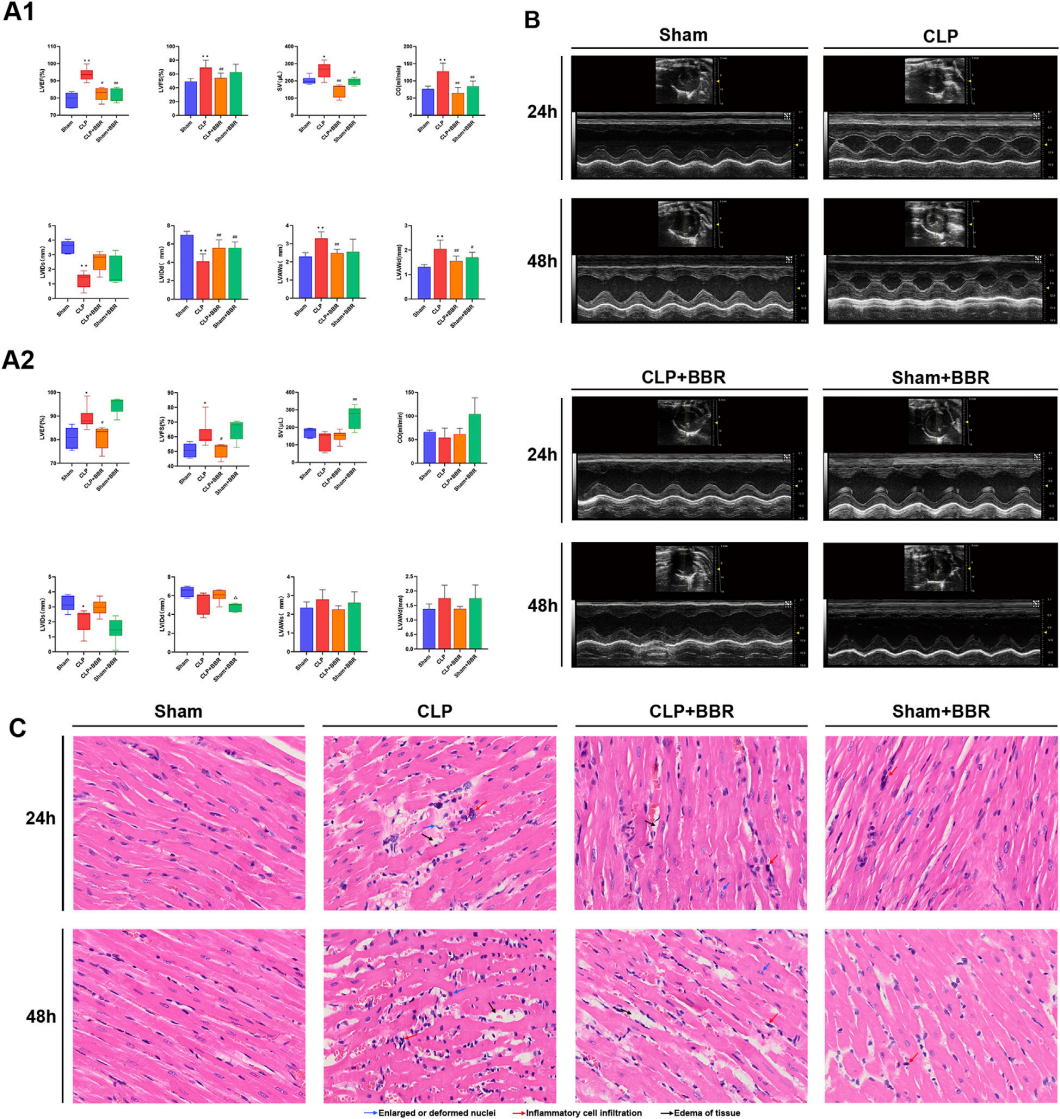
Effects of BBR on Cardiac Function and Myocardial Tissue in Septic Rats **(A1)** Bar graphs illustrating levels of LVEF, LVFS, SV, CO, LVIDs, LVIDd, LVAWs, and LVAWd in rat hearts 24 h post-treatment, as assessed by echocardiography across different groups. **(A2)** Bar graphs demonstrating levels of LVEF, LVFS, SV, CO, LVIDs, LVIDd, LVAWs, and LVAWd in rat hearts 48 h post-treatment, as evaluated by echocardiography for respective groups. **(B)** Representative echocardiograms from each group of rats. **(C)** Histological results of myocardial tissue from different groups stained with Hematoxylin-Eosin (HE). *P < 0.05, **P < 0.01 vs Sham group; ^#^P < 0.05, ^##^P < 0.01 vs CLP group; N = 6, biological replication.

Firstly, we established a rat sepsis-model following CLP modelling and assessed their cardiac function by using echocardiography. The results revealed that 24 h post-modeling, parameters including LVEF, LVFS, SV, CO, LVAWs and LVAWd were significantly elevated in the CLP group compared to the Sham group (Figure 1A1). After BBR intervention was found to decrease LVEF, LVFS, SV, CO, LVAWs, and LVAWd decreased, along with an increase LVIDs and LVIDd increased (Figure 1A1). Interestingly, there was no significant difference observed between the Sham group and the Sham + BBR group (Figure 1A1). After 48 h post-modeling, LVEF and LVFS remained higher in the CLP group than those in the Sham group, accompanied by decreased LVIDs. Additionally, the CLP + BBR group exhibited lower LVEF and LVFS compared to the CLP group (Figure 1A2). The results of echocardiography showed that the abnormally elevated cardiac function was improved, tension and load were reduced, and myocardial damage was alleviated after Ber was given, indicating that the drug inhibited left ventricular shrinkage caused by high cardiac tension and increased left ventricular output per volume and component, thereby improving the ischemic state of myocardial tissues. The effect was more obvious at 24 h than 48 h after modeling, suggesting that Ber could improve cardiac function in the early stage of septic cardiomyopathy (Figure 1B). Histological examination through HE staining showed that the cardiomyocytes were swollen and enlarged, the fibers were broken, the interscalene phase was widened, the myocardial tissue showed red blood cell exudation, and lymphocytes and neutrophils were significantly infiltratedin the CLP group compared with the Sham group, which were ameliorated in the CLP + BBR group (Figure 1C). These findings suggest that BBR intervention improves cardiac function, reduces cardiomyocyte injury, and diminishes inflammatory cell infiltration in septic rats, with a more pronounced effect observed at 24 h post-modeling as compared to 48 h. Overall, BBR demonstrates promising therapeutic potential in attenuating sepsis-induced cardiomyopathy and inflammation, emphasizing the importance of timely intervention for optimal efficacy.

### 3.2 BBR improves mitochondrial structure and function in sepsis rats

**FIGURE 2:**
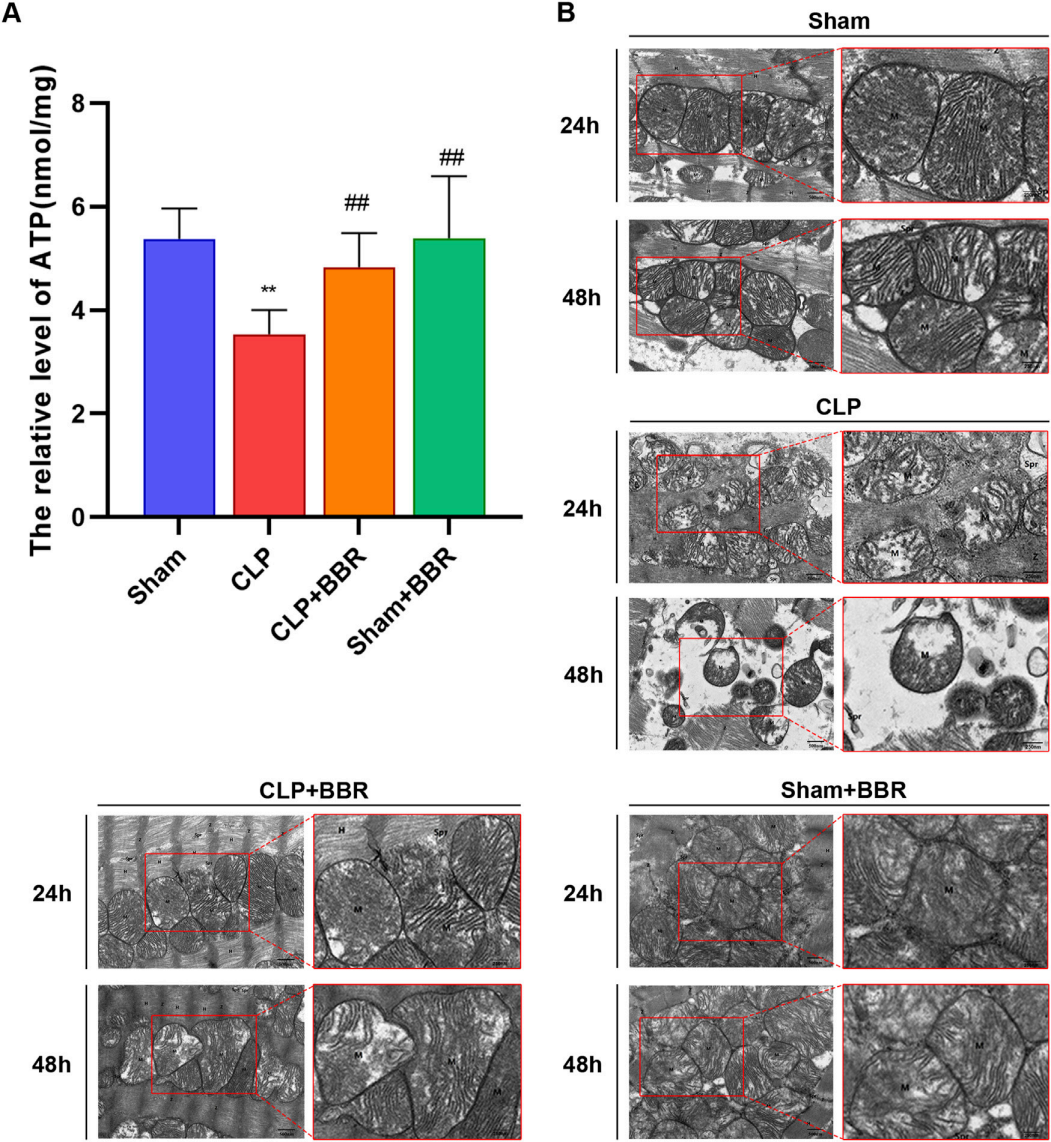
Effect of BBR on mitochondrial function and myocardial tissue in rats with sepsis **(A)** ATP content in myocardial tissue of rats in each group. **(B)** Mitochondrial ultrastructure of myocardial tissue in each group of rats. **P < 0.01 vs Sham group; ^##^P < 0.01 vs CLP group; N = 6, biological replication.

In order to further evaluate the effect of BBR on myocardial mitochondrial function, we detected the ATP concentration in myocardial tissue of rats in each group for 24 h. The results showed that the ATP content of the CLP group (3.53 ± 0.47 nmol/mg) was significantly lower than that of the Sham group (5.38 ± 0.59 nmol/mg), which was partially rescued in the CLP + BBR group (4.83 ± 0.66 nmol/mg) (Figure 2A). Next, we investigated the protective effects of BBR on cardiomyocytes and mitochondria in the rat sepsis model induced by CLP. The electron microscopy results revealed significant structural alterations in cardiomyocytes of the CLP groups compared to the Sham group. Specifically, at 24 h post-CLP, cardiomyocytes exhibited swelling, disorganized myofibrils, and fragmented mitochondria with swollen membranes and decreased cristae density. The severity of cardiomyocyte injury further increased at 48 h post-CLP, characterized by extensive mitochondrial damage and disrupted Z-line and H-band structures. After BBR intervention, the degree of cardiomyocyte and mitochondrial damage was notably improved, with a more pronounced effect seen in the CLP + BBR 24 h group compared to the CLP + BBR 48 h group. There were no significant abnormalities in cardiomyocytes and mitochondria in the Sham group and Sham + BBR group (Figure 2B). Overall, our results indicate that BBR exerts protective effects against sepsis-induced myocardial injury by preserving mitochondrial function.

### 3.3 BBR upregulates the expression of Notch1 signaling in myocardial tissue of sepsis rats

**FIGURE 3:**
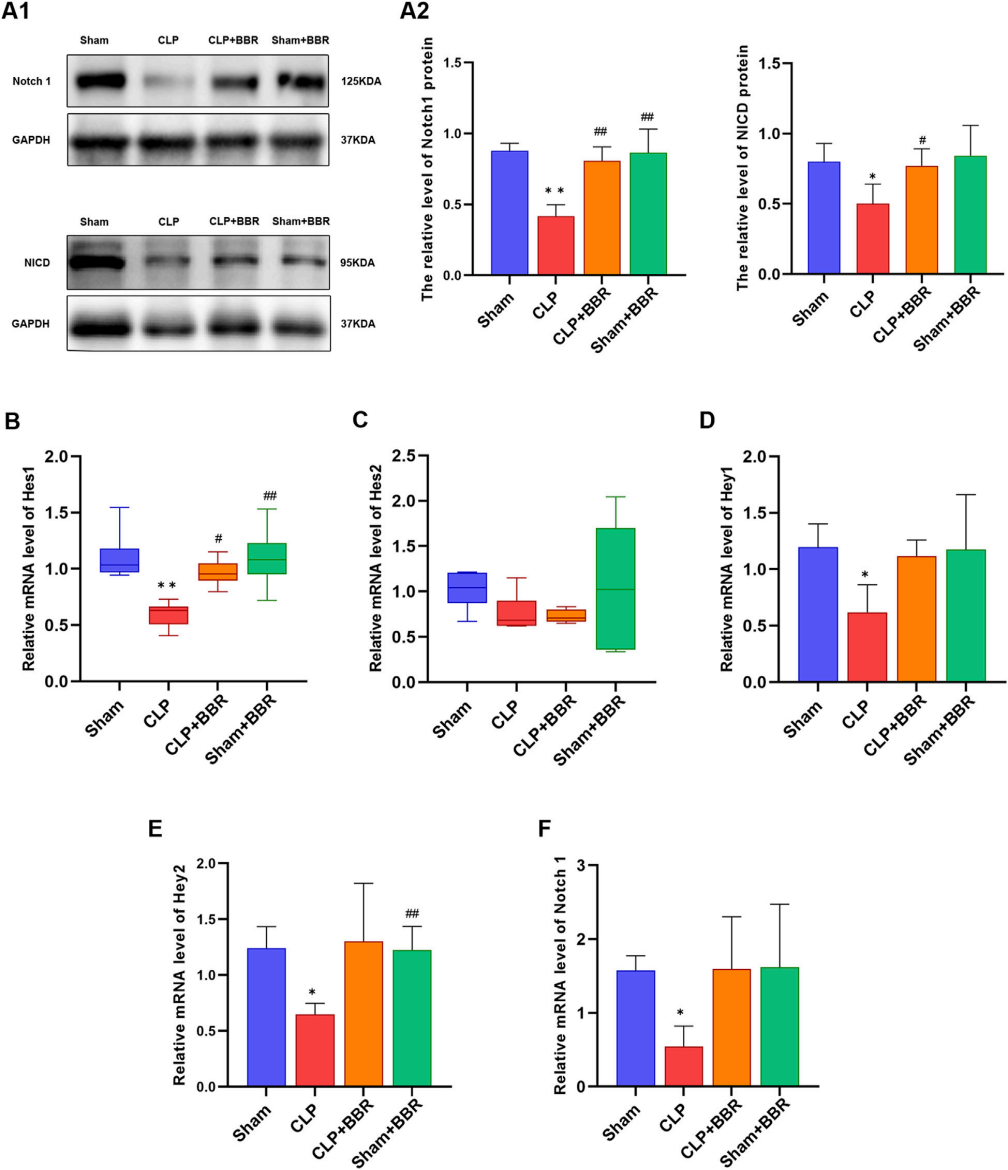
Effect of BBR on Notch1 signaling in myocardial tissue of sepsis rats **(A1)** Western blot of Notch1and NICD in rats in each group. **(A2)** Notch1 and NICD proteins expression level in rats in each group. **(B)** Hes1 gene expression level in rats in each group. **(C)** Hes2 gene expression levels in rats in each group. **(D)** Hey1 gene expression level in rats in each group. **(E)** Hey2 gene expression level in rats in each group. **(F)** Notch1 gene expression level in rats in each group. *P < 0.05, **P < 0.01 vs Sham group; #P < 0.05, ##P < 0.01 vs CLP group; N = 6, biological replication.

Notch is a pivotal regulatory factor in blood vessel formation that controlle organ homeostasis (Sana and Andreas, 2022), involving in heart development during embryogenesis and in the recovery of cardiac function post-myocardial infarction (Olga et al., 2022). Previous study has revealed that Notch1 plays a pivotal role in modulating mitochondrial dynamics, contributes to the maintenance of functional mitochondrial network in cardiac muscle cells (Atsuko et al., 2013). Given the close relationship between Notch1 signaling and mitochondria, we detected the impact of BBR on Notch1 signaling in rats with CLP-induced sepsis. Our findings revealed that the relative expression levels of Notch1 and NICD proteins in myocardial tissue of rats in the CLP group were significantly lower than those in the Sham group, whereas the BBR group exhibited higher expression levels of Notch1 and NICD proteins compared to the CLP group (Figure 3A). To further elucidate the mechanisms underlying this effect, we assessed the expressions of Notch1 protein and mRNA and its downstream genes, including Hes1, Hes2, Hey1 and Hey2 in the myocardial tissues of four groups at 24 h after modeling. Remarkably, we observed that the relative expression levels of Notch1, Hes1, Hes2, Hey1 and Hey2 genes in the CLP group were significantly decreased compared to the Sham group (Figures 3B–F). After BBR intervention, the expression of Hes1 gene increased significantly, and the gene expression of Notch1, whereas Hey1 and Hey2 showed a moderate increase without reaching statistical significance (Figures 3B–F). Collectively, these results demonstrated that BBR potentially alleviated mitochondrial dysfunction via up-regulating Notch1 signaling in sepsis rats.

### 3.4 BBR improved H9C2 cell activity and mitochondrial structure and function

**FIGURE 4:**
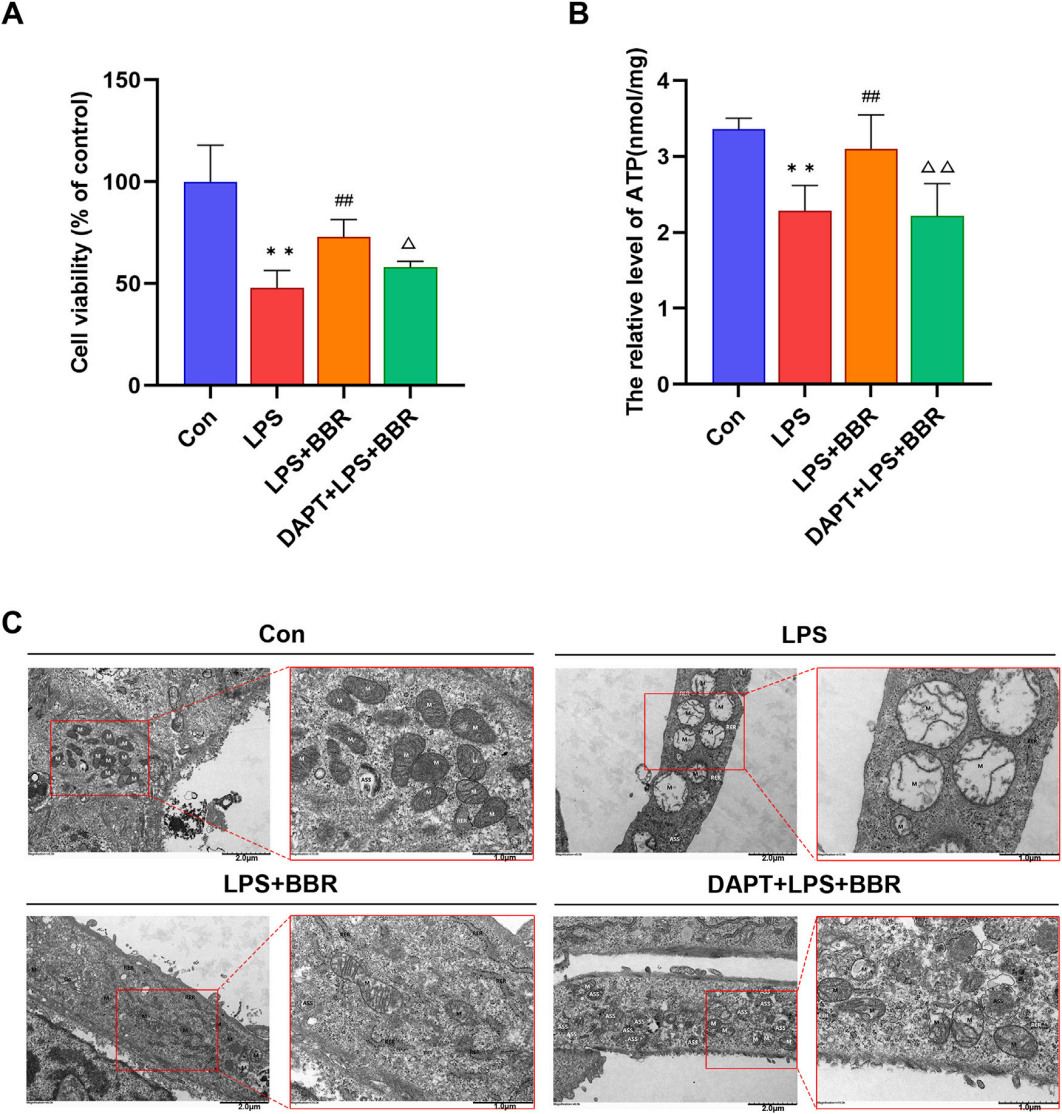
Effects of BBR on H9C2 Cardiomyocyte Viability and Mitochondria **(A)** Viability levels of H9C2 cells across different treatment groups. **(B)** ATP content in H9C2 cells among various groups. **(C)** Ultrastructure of mitochondria in H9C2 cells under different treatments. *P < 0.05, **P < 0.01 vs Con group; ^#^P < 0.05, ^##^P < 0.01 vs LPS group; ^△^P < 0.05, ^△△^P < 0.01 vs LPS + BBR group; N = 6, biological replication.

Next, we explored the impacts of BBR on H9C2 cell viability and mitochondrial integrity. The results of CCK8 assay showed that the activity of H9C2 cells was decreased after LPS induction, with BBR intervention significantly enhancing cell activity compared to in the LPS group. Moreover, inhibition of Notch1 signaling using DAPT (SF4139, Biyuntian, Shanghai, China) resulted a significant decrease in cell activity compared to the BBR group (Figure 4A). Analysis of ATP concentrations further supported the notion that BBR could effectively enhance mitochondrial function in cardiomyocytes, as evidenced by the fact that the LPS + BBR group (3.10 ± 0.18) had a higher ATP concentration than the LPS group (2.28 ± 0.13). Notably, suppression of Notch1 signaling in the LPS + BBR + DAPT group (2.21 ± 0.17) led to a notable reduction in ATP concentration compared to LPS + BBR group (Figure 4B). Subsequent examination via transmission electron microscopy unveiled the overall structure of the cells in the Con group was acceptable. The mitochondria (M) are mostly oval in shape, uniform in size, the membrane is intact, the cristae are mostly parallel and complete, some of the structures are blurred and dissolved, and the matrix is uniform; The rough endoplasmic reticulum (RER) is dilated and less common. The cells in the LPS group were moderately edematous as a whole, and some of them were significantly swollen. The M are swollen and markedly enlarged, the membrane is dissolved in a small area, the cristae are abundantly missing, and the matrix is dissolved in a large area and vacuolated; The RER was not dilated and was short. The cells in the LPS + BBR group were slightly edematous as a whole, and most of them were slightly swollen. The M are uneven in size, some are suspected to be splitting and fusing, the membrane is intact, the cristae are broken and shortened, and the small areas of the matrix are dissolved. The RER was not expanded, and the local area was blurred and fractured. The cells in the LPS + BBR + DAPT group had mild to moderate edema as a whole, and a few had obvious swelling. The M are uniform in size, the local membrane is raised and damaged, the cristae are broken and partially missing, and the small areas of the matrix are dissolved and faded. The RER was not dilated and was short (Figure 4C). Overall, our results demonstrated that BBR could improve the mitochondrial structure damage of H9C2 cells induced by LPS, while DAPT reversed this improvement effect, indicating the ameliorating effect of BBR on the mitochondrial structure damage of H9C2 cells potentially through modulation of Notch1 signaling.

### 3.5 BBR upregulates Notch1 signaling expression in H9C2 cells

To delve into the regulatory role of BBR in Notch1 signaling in H9C2 cells following LPS induction, we detected the expression of the Notch1 and its downstream molecules.

**FIGURE 5:**
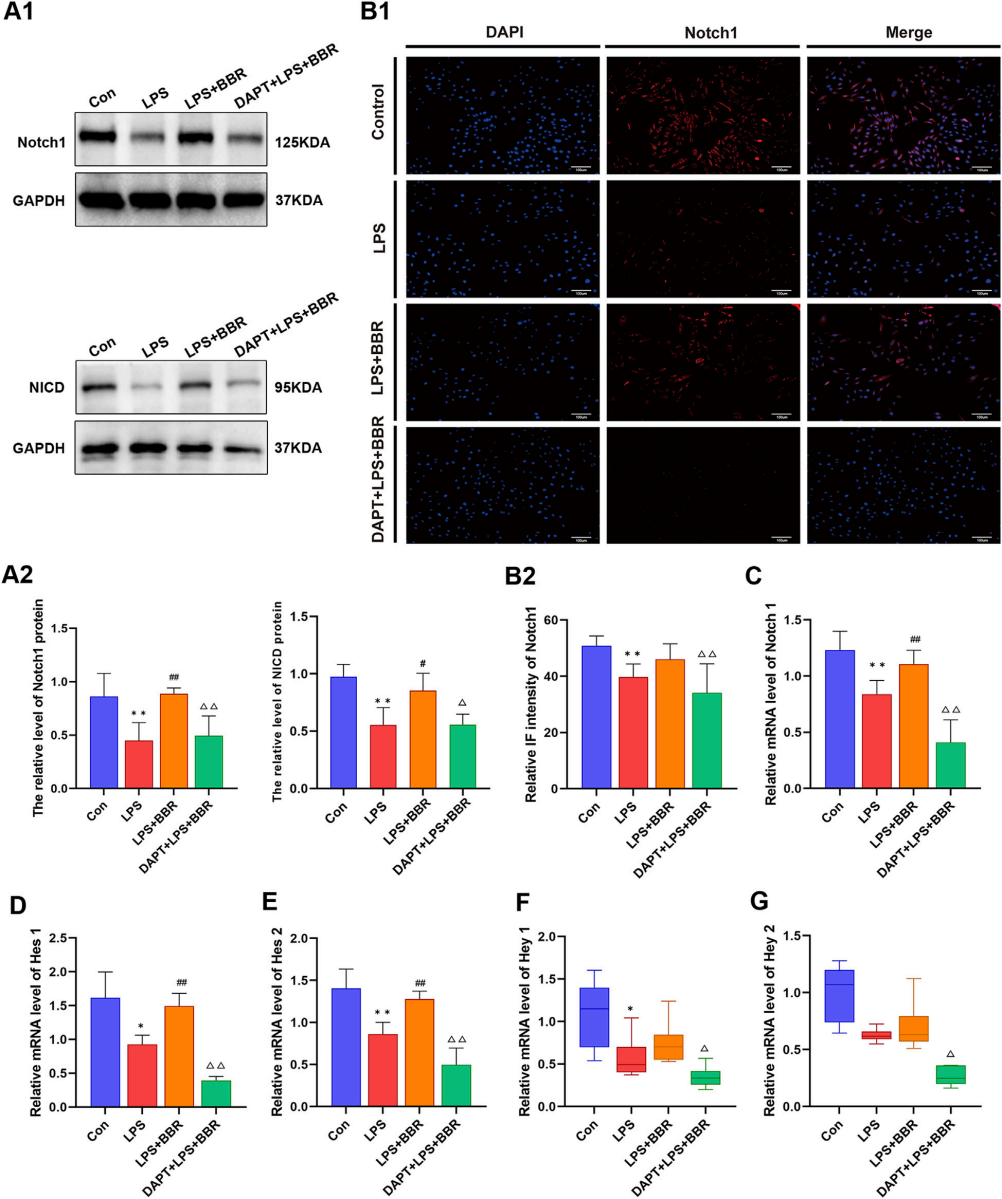
Impact of BBR on Notch1 Signaling in H9C2 Cells **(A1)** Western blot of Notch1 and NICD of cardiomyocytes in each group. **(A2)** Expression levels of Notch1 and NICD proteins in cardiomyocytes in each group. **(B1)** Notch1 immunofluorescence intensity of cardiomyocytes in each group. **(B2)** Expression level of Notch1 immunofluorescence intensity in cardiomyocytes in each group. **(C)** Notch1 gene expression level in cardiomyocytes in each group. **(D)** Hes1 gene expression levels in cardiomyocytes in each group. **(E)** Hes2 gene expression levels in cardiomyocytes in each group. **(F)** Hey1 gene expression levels in cardiomyocytes in each group. **(G)** Hey2 gene expression level in cardiomyocytes in each group. *P < 0.05, **P < 0.01 vs Con group; ^#^P < 0.05, ^##^P < 0.01 vs LPS group; ^△^P < 0.05, ^△△^P < 0.01 vs LPS + BBR group; N = 6, biological replication.

Western blotting results showed that compared with the Con group, the relative levels of Notch1 and NICD proteins were significantly reduced in the LPS group, which were restored upon BBR intervention and subsequently decreased after DAPT inhibition (Figure 5A). Immunofluorescence analysis further supported these findings, demonstrating a decrease in Notch1 immunofluorescence intensity in the LPS group compared to the Con group, with a subsequent increase following BBR intervention and decrease after blocking the Notch1 signal (Figure 5B). RT-PCR data corroborated these observations, the expression level of Notch1 gene in the LPS group was lower than that in the Con group, upregulation after BBR treatment, and downregulation in the LPS + BBR + DAPT group compared to LPS + BBR group (Figure 5C). Additionally, The expression levels of Hes1, Hes2, Hey1 and Hey2 in the LPS group were decrease compared to the Con group, while increase after BBR intervention (Figure 5D–G). Moreover, the gene expression levels of Hes1, Hes2, Hey1 and Hey2 in the LPS + BBR + DAPT group were decreased compared with those in the LPS + BBR group, but the difference was not statistically significant (Figure 5D–G). These comprehensive results collectively suggested that BBR has the capacity to effectively regulate Notch1 signaling in H9C2 cells, involving in cardioprotection and cellular function.

## 4 Discussion

In the current study, our results demonstrated that BBR could significantly reduce myocardial tissue damage, inflammatory cell infiltration, increase the ATP level, and improve the structure and morphology of mitochondria in a rat model of CLP-induced sepsis, indicating that BBR had a protective effect on mitochondrial structure and function in SCM. Additional *in vitro* experiments using H9C2 cells confirmed that BBR could mitigate LPS-induced mitochondrial damage and enhance cell activity through modulation of Notch1 signaling. These findings shed light on the therapeutic potential of BBR in alleviating sepsis-induced cardiac dysfunction and mitochondrial injury.

SCM is characterized by inflammation, myocardial cell injury, microvascular dysfunction, and mitochondrial dysfunction (Ouyang et al., 2022). In this study, a decrease in cardiac contraction and relaxation functions, myocardial cell damage, and infiltration of inflammatory cells was observed in the model group rats, indicating successful construction of the SCM model. Previous study showed that treatment with BBR in the hippocampal tissues of the rats resulted in a decrease in the expression levels of TNF-α, IL-1α and C1qA (Jian et al., 2021). Additionally, our investigation revealed that intervention with BBR could diminished the activation of NF-κB signaling in myocardial cells and decreased the expression levels of TNF-α and IL-1β in myocardial tissues of sepsis rats (Huiqi et al., 2021). Herein, we found that BBR could ameliorate cardiomyocyte injury and reduce inflammatory cell infiltration in myocardial tissue of rats with CLP-induced sepsis, with the efficacy being greater with earlier intervention, aligning with previous research findings (Chen H. et al., 2021).

Extensive downregulation of mitochondrial genes has been observed in the hearts of patients with SCM, accompanied by focal mitochondrial membrane damage, swelling, and alterations in mitochondrial crest structure within cardiomyocytes (Steven and Mervyn, 2021). The progression of these pathological changes ultimately leads to cardiomyocyte death and a marked deterioration in cardiac function (Ying et al., 2019; Michael et al., 2020). Our examination via transmission electron microscopy unveiled distinct cellular morphology alterations across SCM, with cells showcasing mitochondrial swelling and structural damage. Studies have demonstrated that BBR has the ability to regulate mitochondrial functions (Xin et al., 2019; Muyu et al., 2021). What’s more, BBR could modulate mitochondrial respiratory chain function and structure, enhance mitophagy, and mitigate mitochondrial dysfunction, thereby safeguarding cardiac function (Xinyi et al., 2022). Herein, we further delve into whether BBR’s protective effects on SCM rats are linked to mitochondria. Our electron microscopy analysis demonstrates that BBR can ameliorate cardiomyocyte and mitochondrial damage both *in vivo* and *in vitro*, with a more pronounced improvement observed in the 24-hour group compared to the 48-h group, revealing a significant enhancement by BBR on cardiac mitochondrial structure and function.

Research demonstrated that Notch1 signaling provides protective effects against conditions such as cardiomyopathy, myocardial infarction, pathological cardiac enlargement, and damage caused by ischemia-reperfusion (Meester et al., 2018). What’s more, Notch signaling has been shown to play a cardioprotective role by inhibiting ROS production and stabilizing mitochondrial membrane potential after myocardial injury (Luxán et al., 2015). Notch signaling also influences mitochondrial activity through its interactions with the electron transport chain (Jiewen et al., 2022). Significantly, the results of our cell experiments showed that blocking the Notch signaling pathway led to decreased cell viability, damaged mitochondrial structure, decreased ATP levels, and impaired mitochondrial function. More importantly, DAPT, an inhibitor of Notch signaling pathway could weaken the protective effect of BBR on cardiomyocytes and mitochondrial structure. Upon binding of the Notch receptor to its ligand, the receptor undergoes cleavage by gamma secretase enzymes, leading to the release of the Notch intracellular domain (NICD), which translocates to the nucleus, binds to the transcription factor RBP-Jκ, activates downstream target genes (Hes and Hey), and initiates the transduction of the Notch signaling pathway (Stefano and Ernesto, 2016). Further detection of Notch1 receptor protein and its downstream signaling molecules in myocardial tissues and cells showed that BBR’s intervention could increase the expression level of Notch1 receptor in SCM rats. Through a comprehensive experimental design, we systematically evaluated the role of berberine (BBR) at multiple levels, especially in mitochondrial function, providing a more comprehensive and in-depth understanding.

## 5 Conclusion

In summary, our study confirms the pharmacological effects of berberine (BBR) on septic cardiomyopathy (SCM) and further identifies its multifaceted regulatory roles in mitochondrial function and inflammatory responses. These mechanisms, combined with the activation of the Notch signaling pathway, highlight the pharmacokinetic characteristics and therapeutic potential of BBR in SCM. BBR has a flat, rigid structure, and nitrogen atoms are positively charged, properties that contribute to the ability to interact with multiple targets and pathways (Ai et al., 2021). BBR’s fat-soluble properties allow it to cross the cell membrane and enter the interior of the cell, interacting with key molecules of the Notch1 signaling pathway. Compared to previous studies that separately focused on NOTCH, mitochondria, and septic cardiomyopathy (Liu et al., 2022; Chang et al., 2021; Tan et al., 2019), our findings highlight the characteristic multi-target action of traditional Chinese medicine. Specifically, our results suggested that BBR can improve SCM by protecting mitochondrial function in cardiomyocytes via upregulation of Notch1 signaling, and by inhibiting inflammation to exert anti-inflammatory effects and reduce myocardial injury. This study provides a new idea for the clinical application of BBR in the future and the development of targeted therapy for SCM. Despite the promising findings, our investigation also reveals several avenues for future research. Particularly, there remains a crucial need to conduct additional experiments to comprehensively assess mitochondrial dynamics. Furthermore, a more thorough examination of how BBR modulates the Notch1 signaling pathway is essential. Extensive genetic studies, such as employing gene knock-out models that target the Notch pathway, are essential to precisely delineate the molecular pathways affected by BBR. These aspects will be explored in our further study. Of course, the mechanism of septic cardiomyopathy is complex, and translating laboratory research into clinical applications is a complex task that involves multiple challenges, including dose optimization, selection of administration routes, and development of personalized treatment plans, which still require further research.

## 6 Scope statement

Septic cardiomyopathy (SCM) is a significant complication arising from sepsis-related cardiovascular dysfunction, yet there is currently no targeted therapy available. This manuscript investigates the therapeutic effects of berberine (BBR) on SCM, aiming to elucidate its underlying mechanisms of action. Using a septic cardiomyopathy rat model established via cecal ligation and puncture (CLP) and an H9C2 cardiomyocyte injury model induced by lipopolysaccharide (LPS), we assessed cardiac function through echocardiography and examined myocardial histopathology with hematoxylin-eosin staining. Our findings demonstrate that BBR significantly improves cardiac function and mitochondrial structure in SCM rats, enhances ATP levels, and upregulates Notch1 signaling pathways. *In vitro*, BBR increased H9C2 cell viability and improved mitochondrial morphology while promoting the expression of Notch1 and its downstream targets. Notably, these beneficial effects were reversed upon inhibition of Notch1 signaling, highlighting its critical role in BBR’s mechanism of action. This study contributes to the understanding of SCM management and offers insights into potential therapeutic strategies, aligning with the journal’s focus on cardiovascular research and innovative treatment approaches.

## Data Availability

The original contributions presented in the study are included in the article/supplementary material, further inquiries can be directed to the corresponding author.

## Abbreviations

SCM, septic cardiomyopathy; Ber, berberine; CLP, cecum ligation and puncture; LPS, Lipopolysaccharides; LVEF, Left Ventricular Ejection Fractions; LVFS, Left Ventricular Fractional Shortening; SV, Strake volume; CO, Cardiac output; LVAWs, Left ventricular anterior wall thickness in systole; LVAWd, Left ventricular anterior wall thickness in diastole; LVIDs, Left ventricle internal diameter in systole; LVIDd, Left ventricle internal diameter in diastole; ATP, Adenosine triphosphate; IL-1, Interleukin-1; IL-6, Interleukin-1; TNF-α, Tumor Necrosis Factor-α; TACE, TNF-α converting enzyme; NICD, Notch Intracellular Domain; RBP-Jκ, recombination signal binding protein for immunoglobulin kappa J region; NF-κB, Nuclear factor kappa-B; NLRP3, NOD-like receptor thermal protein domain associated protein 3; Drp1, Dynamin-related protein 1; PGC-1α, peroxisome proliIerators-activated receptor γ coactivator lalpha
